# Ambulance Tongas from India for South Africa

**Published:** 1900-08-11

**Authors:** 


### AMBULANCE TONGAS FROM INDIA FOR SOUTH AFRICA.

The very acceptable donation of twenty ambulance tongas or carts, fully equipped, was made to our troops in South Africa by Mr. Dhanjibhoy, C.I.E., of Rawul Pindi, in the Punjab. Tongas, we may explain, are the light carts used for the conveyance of mails and passengers in the hills of Northern India. They are low-hung, and in form much resemble a child's mail cart, with the addition of a hood and the substitution of a pole and curricle bar for the shafts. Mr. Dhanjibhoy's experience as the proprietor of the largest mail cart lines in the Punjab has enabled him to devise a form of tonga for the conveyance of the sick and wounded in the field, which has been tested and found eminently satisfactory both on service in Tirah and at various camps of exercise in India. As the conditions of service in South Africa are not dissimilar to those of Northern India, there is every reason to anticipate that the tongas will prove equally useful in the Transvaal. Lord Roberts has already telegraphed his thanks to Mr. Dhanjibhoy and his approval of the form of ambulance.

Mr. Dhanjibhoy's tonga train comprises fifty horses and twenty vehicles, twelve of which are large with stretchers and eight small without stretchers. The tongas are hung upon springs to minimise shaking, and can be used with or without the stretchers. When used without the stretchers they accommodate two in a recumbent position, or if the patients are capable of sitting up the larger pattern can conveniently hold five, and the smaller three, wounded men, and there is ample space for surgical instruments, pouches, kits, &c., and fittings for a carbine or rifle. The stretcher, when not in use, can, with any heavy baggage, be secured to the splashboard. There are waterproof curtains on all four sides to protect the occupants from the weather, and two of the tongas are fitted with sufficient waterproof material to form a *tente d'abri* when halted. Each tonga is provided with a leather water bag containing two gallons. An ingenious addition of a spring to the curricle bar obviates almost entirely the noise and jolting which generally accompanies this description of draught.

Tested at a recent camp of exercise, this form of tonga was reported on by the principal medical officer as being successful in every way, and affording a ready means for the removal of wounded from the artillery and cavalry. "It was quite capable of keeping up with the artillery, and was on the ground with cavalry almost as soon as its services were required, though it could not quite keep pace with this arm." "It appears to be a most valuable cart for rendering aid to the wounded owing to the great rapidity with which it can be moved and its easy motion."

Parsees are well-known in India for their generosity and philanthropy, and the recent gift of Mr. Dhanjibhoy to our troops is but another example of the munificent donations we owe to this race.

